# Proteomic profiling and functional analysis of extracellular vesicles from metastasis-competent circulating tumor cells in colon cancer

**DOI:** 10.1186/s13046-025-03360-4

**Published:** 2025-03-22

**Authors:** Luis Enrique Cortés-Hernández, Zahra Eslami-S, Aurore Attina, Silvia Batista, Laure Cayrefourcq, Jérôme Vialeret, Dolores Di Vizio, Christophe Hirtz, Bruno Costa-Silva, Catherine Alix-Panabières

**Affiliations:** 1 Laboratory of Rare Human Circulating Cells, University Medical Center of Montpellier, Montpellier, France; 2 CREEC, MIVEGEC, University of Montpellier, CNRS, IRD, Montpellier, France; 3 IRMB-PPC, INM, Univ Montpellier, CHU Montpellier, INSERM CNRS, Montpellier, France; 4 Systems Oncology Group, Champalimaud Centre for the Unknown, Lisbon, Portugal; 5 Department of Urology, Division of Cancer Biology and Therapeutics, Cedars-Sinai Medical Center, Los Angeles, CA USA; 6 European Liquid Biopsy Society (ELBS), Hamburg, Germany

**Keywords:** Extracellular vesicles, Circulating tumor cells, Colorectal cancer, Proteome, Cancer progression, Liquid biopsy

## Abstract

**Background:**

Circulating tumor cells (CTCs) are pivotal in cancer progression, and in vitro CTC models are crucial for understanding their biological mechanisms. This study focused on the characterization of extracellular vesicles (EVs) from CTC lines derived from a patient with metastatic colorectal cancer (mCRC) at different stages of progression who progressed despite therapy (thus mirroring the clonal evolution of cancer).

**Methods and results:**

Morphological and size analyses revealed variations among EVs derived from different CTC lines. Compared with the Vesiclepedia database, proteomic profiling of these EVs revealed enrichment of proteins related to stemness, endosomal biogenesis, and mCRC prognosis. Integrin family proteins were significantly enriched in EVs from CTC lines derived after therapy failure. The role of these EVs in cancer progression was analyzed by assessing their in vivo distribution, particularly in the liver, lungs, kidneys, and bones. EVs accumulate significantly in the liver, followed by the lungs, kidneys and femurs.

**Conclusions:**

This study is a pioneering effort in highlighting therapy progression-associated changes in EVs from mCRC patients via an in vitro CTC model. The results offer insights into the role of metastasis initiator CTC-derived EVs in cancer spread, suggesting their utility for studying cancer tissue distribution mechanisms. However, these findings must be confirmed and extended to patients with mCRC. This work underscores the potential of CTC-derived EVs as tools for understanding cancer dissemination.

**Supplementary Information:**

The online version contains supplementary material available at 10.1186/s13046-025-03360-4.

## Background

Advances in in vitro cell expansion techniques have provided an opportunity to analyze circulating tumor cells (CTCs) precisely and incorporate a temporal dimension into the study of cancer progression [1]. We successfully established a unique series of CTC lines derived from patients with metastatic colorectal cancer (mCRC). These colon CTC lines represent the clonal evolution of cancer cells during disease progression and therapy, including the development of therapy resistance [2]. Cayrefourcq et al. demonstrated that these CTC lines clearly differ in gene expression [2]. For example, the number of upregulated genes related to the mTOR and PI3K/AKT pathways was significantly increased in CTC lines obtained after therapy failure [3]. Additionally, genes related to xenobiotic metabolism, such as cytidine deaminase, an enzyme used by cancer cells to metabolize 5-fluorouracil (5-FU), a standard chemotherapy drug for CRC, are upregulated [2].

In cancer cells, the development of resistance to therapeutic agents involves several pathways and cellular mechanisms that are interconnected with cancer progression, including tumor expansion and metastasis. For example, it has been proposed that the secretion of extracellular vesicles (EVs) is involved in cancer progression associated with CTC organotropism [4, 5]. The presence of different kinds of surface proteins, such as integrins, results in specific organ distributions in vivo and further changes associated with premetastatic niche formation. Moreover, it has also been recently reported that the liver metabolism of drugs is influenced by tumor EVs and particles (e.g., exomeres) [6].

EVs and CTCs express similar surface proteins that are required for interaction with microvessels during intravasation. EVs might also present changes in protein composition during cancer progression under treatment [7]. These studies point to a possible role of EVs in cancer progression despite therapy. However, no study has demonstrated the association of tumor progression with changes in the EV proteome or the impact of EVs on cancer progression and metastasis mechanisms. EVs and CTCs express similar surface proteins (i.e., integrins) that are required for interaction with microvessels during intravasation. EVs from in vitro-expanded CTCs could be used to explore changes in the EV proteome at different steps during cancer progression. This could allow the identification of protein markers in EVs to be used for monitoring therapy failure via liquid biopsy. In this work, we analyzed the proteome of EVs released by metastasis-competent CTC lines derived from the same patient at various stages of mCRC progression and therapy failure. We then investigated the biodistribution of these EVs in vivo.

## Methods

### Colon CTC lines

CTC lines were established in our laboratory [8, 9] from a patient with mCRC under 5-FU and platinum-based therapy before treatment initiation (CTC-MCC-41), after second relapse and second-line treatment (CTC-MCC-41.4), and after the third relapse/shortly before death (CTC-MCC-41.5G). These cell lines were cultured in suspension in RPMI 1640 (Corning 15363561, US) supplemented with 10% fetal calf serum (Eurobio™, CVFSVF00-01), 1% insulin-transferrin-selenium (Gibco™ ITS-G 41400045, US), epithelial growth factor (20 ng/mL, Milteny Biotec™, 130-093-825, Germany), L-glutamine (2 mM, Eurobio™, CSTGLU00_0U), and fibroblast growth factor-2 (10 ng/mL, Milteny Biotec™, 130-093-840, Germany) at 5% CO_2_ and 37 °C. The CTC-MCC-41 line was transfected with a lentivirus probe expressing Luc/mCherry. CTC-MCC-41-Luc/mCherry cells were cultured under the same conditions as the other CTC lines were cultured.

The SW480 (primary CRC, ATCC SW-480 CCL-228™) and SW620 (mCRC, ATCC SW-620 CCL-227™) cell lines were used as controls and cultured under adherent conditions with Dulbecco’s modified Eagle’s medium (Life Technologies, 31966 US) and 10% fetal bovine serum (FBS) (Biowest S181BH-500; Nuaillé, France).

All the cell lines were conditioned (for 24 h) before EV isolation via the usual culture medium, but with FBS depleted of bovine EVs by ultracentrifugation at 100,000 × g for 140 min.

### EV isolation

For EV isolation, conditioned medium was centrifuged first at 500 × g for 10 min and then at 3,000 × g for 20 min to eliminate suspended or dead cells. To remove large EVs, the medium was centrifuged at 12,000 × g for 20 min, and the resulting pellet was discarded. The supernatant, which contained EVs, was centrifuged again at 100,000 × g for 140 min for EV enrichment, and the pellet was collected.

For purification, the EV pellet was resuspended in 14 ml of sterile phosphate-buffered saline (PBS) that had been filtered through a 0.22 μm membrane. Then, the EVs were gently layered on top of a 4 ml sucrose mixture consisting of deuterium oxide (D_2_O), protease-free sucrose (1.2 g), and Tris base (96 mg) adjusted to pH 7.4. After ultracentrifugation at 100,000 × g for 70 min, 4 ml of the sucrose fraction was carefully collected from the bottom of the ultracentrifugation tube via an 18G needle, away from the pellet. Then, 16 ml of sterile PBS was added to the collected sucrose/EV mixture, followed by overnight ultracentrifugation at 100,000 × g for 16 h. Finally, the pellet containing the isolated EVs was resuspended in filtered PBS.

All the solutions used, including PBS and the sucrose cushion, were sterile and filtered through a 0.22 μm membrane. All centrifugation steps were performed at 10 °C using a 70Ti rotor (Beckman-Coulter™, US).

The cell viability before and during conditioning with EV-depleted FBS was > 90% before EV isolation, and all the cultures were confirmed to be mycoplasma free.

### EV characterization

The initial EV characterization was carried out following the recommendations outlined in the International Society for Extracellular Vesicles (ISEV) guidelines [10]. An NS300 nanoparticle tracking analysis (NTA) system with a red laser (638 nm) from NanoSight (Malvern Panalytical©, United Kingdom) was used to determine the EV concentration and size distribution. The settings for the analysis were as follows: video acquisitions with a camera level set at 16 and a threshold between 5 and 7. For each sample, video acquisition lasted 30 s, and five videos were recorded. The NTA software version 3.4 (Malvern Panalytical©, United Kingdom) was used for the analysis. The protein content of the EV samples was subsequently determined via a BCA assay (Pierce TM BCA Protein Assay Kit™, Thermo Fisher Scientific©).

To assess their morphology, EVs were fixed in 2% paraformaldehyde and stained with filtered 1% uranyl acetate. Imaging was performed via a Veleta (2Kx2K) CCD camera coupled to a Tecnai F20 electron microscope at acceleration voltages ranging from 80 kV to 100 kV. Ten to twenty field images were obtained, and an EV was defined on the basis of the classical cup-shaped morphology [10].

### EV proteomic characterization

Mass spectrometry experiments were carried out at the Montpellier Proteomics Platform (PPM-PPC, BioCampus Montpellier). For each EV sample, 35 µg of protein, measured via the BCA assay (Pierce TM BCA Protein Assay Kit™, Thermo Fisher Scientific©), was used for analysis. The samples were dried in a Speedvac (Labconco, Kansas, USA) and then mixed as follows: 19.5 µL of sample, 7.5 µL of NuPage LDS (4x) (Thermo Fisher©), and 3 µL of the reducing agent dithiothreitol (DTT) (10x). The samples were subsequently separated on NuPAGE 4–12% gels (Thermo Fisher©) and stained with SimplyBlue™ SafeStain (Thermo Fisher©). Each gel was cut into eight pieces via a scalpel. The gel pieces were covered with 10 mM DTT and 50 mM ammonium bicarbonate (ABC) (pH 8.4) for 20 min. The DTT solution was then removed, and 150 µL of 50% ethanol/50 mM ABC (pH 8.4) was added to each gel. The alkylation step was performed using a solution containing 55 mM 2-iodoacetamide and 50 mM ABC (pH 8.4). To dehydrate the gel pieces, 100 µL of 50% ethanol/50 mM ABC (pH 8.4) was added, followed by trypsinization of the dry gel pieces at 37 °C and 450 rpm overnight. For the Liquid Chromatography-Ultrahigh Resolution-Quadrupole Time of Flight-tandem Mass Spectrometry (LC-UHR-QqTOF-MS/MS) analysis, samples were processed for label-free quantification (LFQ) with a UHR-QqTOF mass spectrometer (Impact II™, Bruker Daltonics©) as follows: 7 µL of sample was injected into a nanoRSLC Elute apparatus (Bruker Daltonics©). NanoFlow LC was coupled to a QTOF MS instrument (Impact II™, Bruker Daltonics©) through a captive spray ion source operating with a nanobooster. In the LC part, the samples were desalted and preconcentrated online on a PepMap µ-precolumn (300 μm × 5 mm, C18 PepMap 100, 5 μm, 100 Å). The peptides were transferred to an analytical column (75 μm × 500 mm; Acclaim Pepmap RSLC, C18, 2 μm, 100 Å) for separation. A gradient consisting of 7–30% B for 45 min and 90% B for 4 min (A = 0.1% formic acid, 2% acetonitrile in water; B = 0.1% formic acid in acetonitrile) at 400 nL/min and 50 °C was used to elute peptides from the reverse-phase column.

Data-dependent acquisition was performed to identify peptides, and a lock-mass (m/z 1222, Hexakis (1 H, 1 H, 4 H-hexafluorobutyloxy) phosphazine) was used as an internal calibrator. The Instant Expertise software selected the most intense ions per cycle of 3 s, and active exclusion was performed after 1 spectrum for 2 min unless the precursor ion exhibited an intensity higher than 3 compared with the previous scan.

### Proteomic database search analysis

LC-UHR-QqTOF-MS/MS data were processed via MaxQuant (v1.6.17.0; MaxPlank, Germany). All the data were queried against the UniProt complete human proteome via the following parameters: Enzyme Trypsin/P, Maximum allowed missed cleavage sites 2, Main search peptide tolerance 10 ppm, Isotope match tolerance 0.005 Da, Dynamic modifications Oxidation (M), Acetyl (Protein N-term), and Static modification Carbamidomethyl (C). Percolator was used to calculate the peptide false discovery rates (FDRs) per file. An FDR of 2.5% was applied to each separate file. The protein search database was then searched with Perseus (v1.6.15.0, MaxPlank, Germany), LFQ-Analyst [11] and FunRich [12]. For LFQ, missing values were imputed via the BPCA imputation method. A cut-off of the adjusted *p*-value of 0.05 (Benjamini-Hochberg method) along with a log2 fold change of 1 has been applied to determine significantly regulated proteins in each pairwise comparison, and a Log fold change cut-off ≥ 0.6.

### Multiple reaction monitoring (MRM) panel

Seventeen proteins (differentially expressed in EVs from CTC lines) were selected from the peptides detected via LFQ. For these proteins, 36 peptides (2 or 3 per protein) were selected for the MRM assay. The list of synthetic peptides (Aqua HeavyBasic™, Thermo Scientific ©) is provided in Table S1.

Twenty-five micrograms of protein were used per EV sample: 6 replicates for CTC-MCC-41 EVs, 6 replicates for CTC-MCC-41.4 EVs, and 8 replicates for CTC-MCC-41.5G EVs (replicates were from different batches). The samples were processed without 1D-gel SDS‒PAGE separation, following the same protocol of denaturation, reduction, alkylation and trypsinization described in Sect. 2.4. The synthetic peptides were cleaned with C18 tips (Agilent Technologies, Santa Clara, USA). Before sample loading, C18 tips were primed with 70% acetonitrile/0.1% trifluoracetic acid (TFA) and equilibrated with 0.1% TFA. The digested sample clean-up included washing with 0.1% TFA followed by elution with 70% acetonitrile/0.1% TFA. Clean peptides were dried in a Speedvac (Labconco, Kansas, USA). The samples were resuspended in 10 µL of 2% acetonitrile/0.1% formic acid/97.9% water by stirring for 10 min before LC‒MS/MS analysis.

The samples were processed with a UHR-QqTOF mass spectrophotometer (Impact II™, Bruker Daltonics©, US). The data were analyzed with Perseus (v1.6.15.0, MaxPlank, Germany).

### EV biodistribution assay

The biodistribution assay was performed in triplicate using the same batch of EVs from all cell lines (CTC-MCC-41, CTC-MCC-41.4, CTC-MCC-41.5G, SW480 and SW620) for proteomic analysis. The total EV amounts were normalized according to the number of particles and total amount of protein to obtain a minimum protein concentration of 5 µg per injection. Then, the EVs were labeled with the CellVue™ Burgundy Cell Labeling Kit (Invitrogen™, 8-0872-16) according to the manufacturer’s instructions. EVs (or the PBS control) were injected via the retroorbital plexus in 6- to 8-week-old NOD-SCID mice (*n* = 25, 5 mice for each condition). After 24 h, the mice were sacrificed, and the organs (femur, brain, lungs, kidneys, intestine and liver) were recovered. EV distribution in the organs was visualized via the Odyssey™ DLx imaging system (Li-COR Biosciences©, Germany). The data were analyzed with Image Studio™ software (Li-COR Biosciences©, Germany). All animal experiments were approved by the Champalimaud Foundation Animal Welfare Body (Protocol number 2017/006).

### Statistical analysis

When needed, statistical significance was assessed via ANOVA or the Kruskal‒Wallis test. *Post hoc* analysis was subsequently performed. *P* values < 0.05 were considered significant. Statistical analyses were performed via R version 4.3.3 (R-project.org) and Biorender^®^ (BioRender.com).

## Results

### EV characteristics vary among colon CTC lines derived at different stages of disease progression

After isolation, the initial analysis revealed a predominance of particles within the EV size range (Figure S1**a**). Morphological characterization by electron microscopy confirmed the classically described “cup-shaped” structure of the EVs in all the samples, suggesting the successful isolation of the EVs (Figure S1**b**). The mean sizes of the particles isolated from the medium of the CTC lines CTC-MCC-41, CTC-MCC-41.4 and CTC-MCC-41.5G were 126 nm, 127 nm, and 109 nm, respectively. EVs from CTC-MCC-41.5G cells presented the highest ratio of particles per microgram of protein and the highest particle count (Figure S1**c**).

**Fig. 1:**
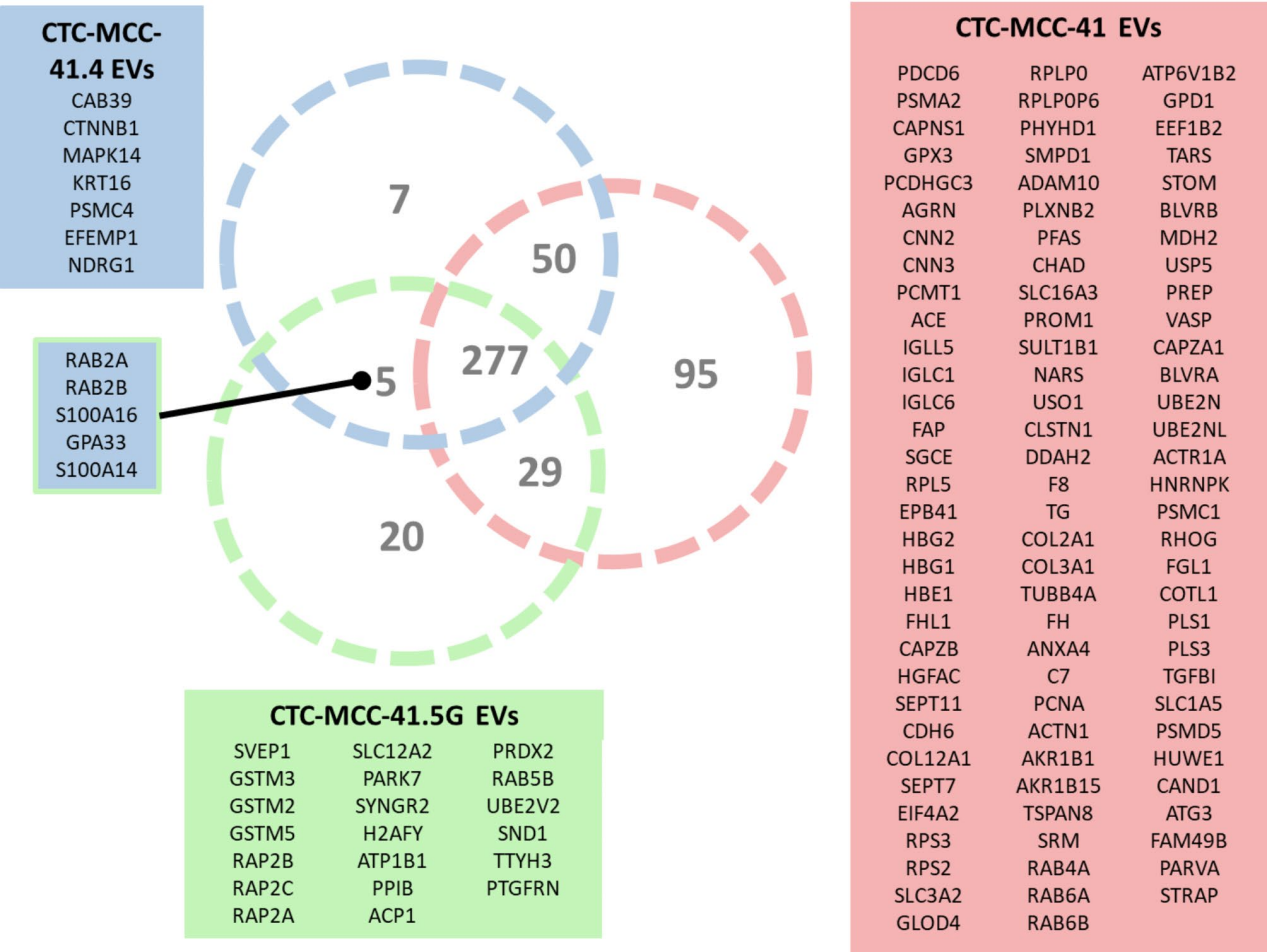
Presence-absent analysis of the EV proteomic cargo. The EVs from the CTC lines presented 277 shared proteins. The analysis also identified 95 unique proteins in EVs from CTC-MCC-41 cells, 7 in EVs from CTC-MCC-41.4 cells, and 20 in EVs from CTC-MCC-41.5G cells. EVs from CTC-MCC-41.4 and 41.5G shared most of proteins, in contrast to CTC-MCC-41 EVs, these EVs showed the highest number of unique proteins

Proteomic analysis revealed 480 proteins in EV samples; 95 unique proteins were identified in EVs derived from CTC-MCC-41 cells, 7 in EVs derived from CTC-MCC-41.4 cells, and 20 in EVs derived from CTC-MCC-41.5G cells (Fig. 1). Overall, 277 proteins were commonly identified across EVs from all three CTC lines (Fig. 1). Notably, only five proteins were shared between EVs from CTC-MCC-41.4 and CTC-MCC-41.5G. Among these groups, EVs from CTC-MCC-41.4 and CTC-MCC-41.5G presented the smallest proportion of shared proteins (64.3%) (Figure S1**d**).

**Fig. 2:**
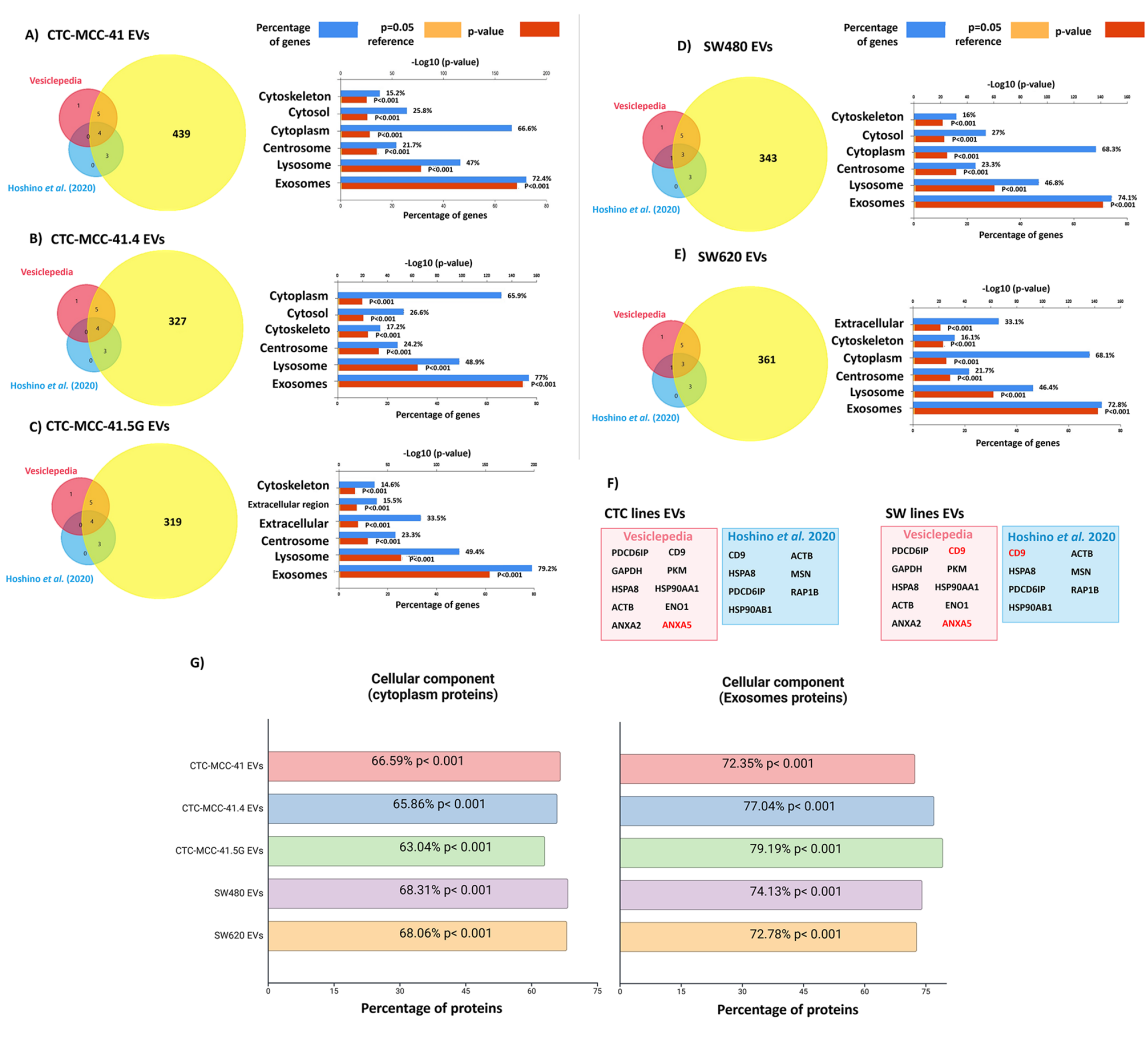
Cellular component analysis of the EV proteomic cargo based on present-absent analysis. **A**) to **E**) Comparison of the EV proteins identified with those listed in Vesiclepedia and those identified by Hoshino et al. (2020). **F)** ANXA5 was not detected in EV preparations from the three CTC lines or the two colon cancer cell lines, and CD9 was absent only in SW480 and SW620 cell-derived EVs. **G)** Cellular component enrichment in the indicated EV samples according to the Vesiclepedia data for “Cytoplasm” and “Exosomes”, revealing significantly greater enrichment of “Exosome”-associated proteins in EVs from CTC-MCC-41.4 and CTC-MCC-41.5G cells (derived after therapy failure) than in those from the other colon cancer cell lines

To confirm the presence of proteins previously reported to be associated with EVs, the identified EV proteins were compared with the ten most commonly reported EV proteins according to Vesiclepedia [13] and the seven most frequent proteins reported by Hoshino et al. [14]. Most of these proteins were detected in EVs from all three CTC lines, with the exception of ANXA5, and in EVs from the two CRC lines, with the exception of CD9 and ANXA5 (Fig. 2a-f).

**Fig. 3:**
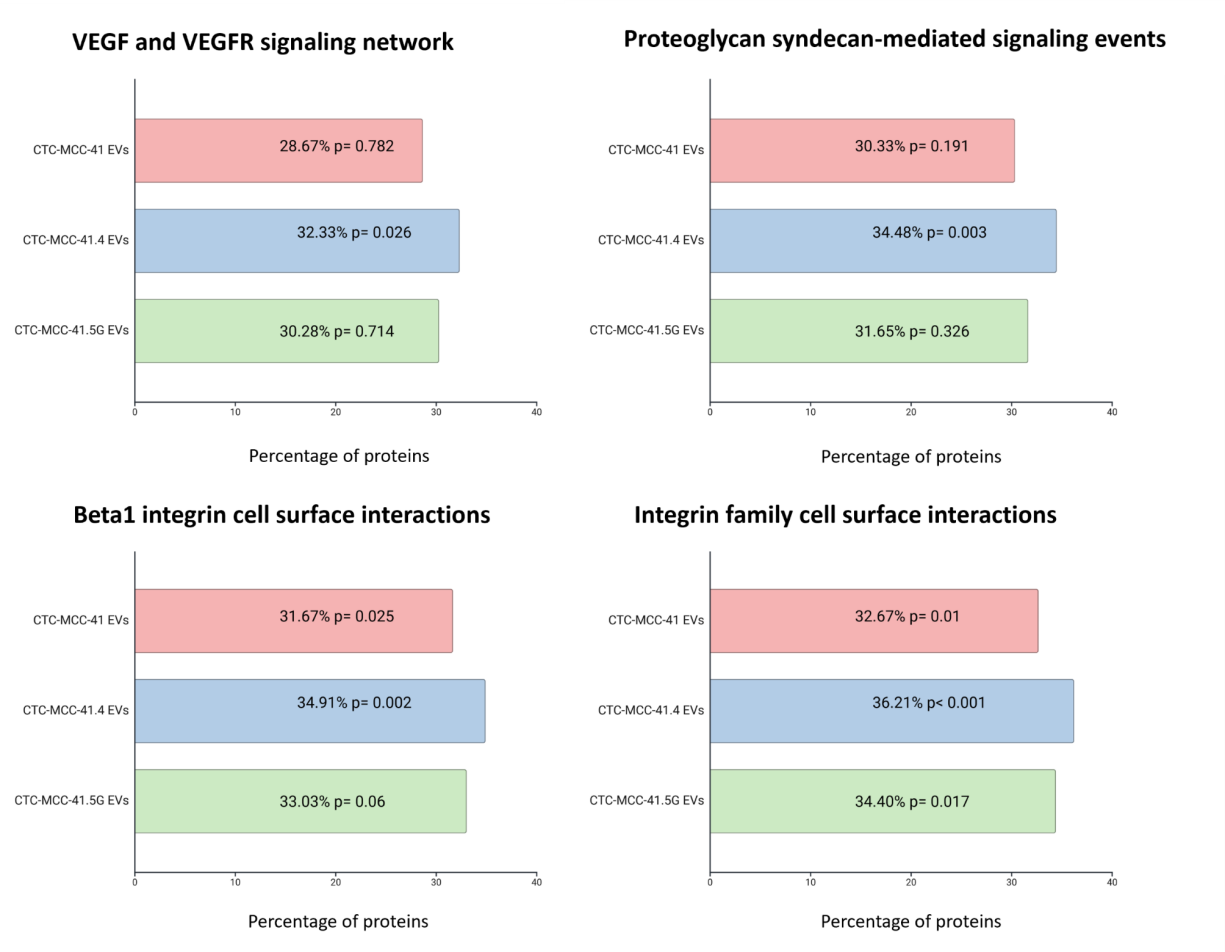
Pathway analysis of proteins identified in EVs. Proteins related to interactions with the integrin family were significantly enriched in EVs from the three CTC lines, specially in EVs from the CTC-MCC-41.4 and 41.5G

Compared with the different cellular components reported in Vesiclepedia [13] (Fig. 2g), the “exosome” component was significantly more common than the “cytoplasmic” component in all EV preparations. This effect was more pronounced in EVs from CTC-MCC-41.4 (77.04%, *p* < 0.001 vs. 65.68%, *p* < 0.001) and CTC-MCC-41.5G cells (79.19%, *p* < 0.001 vs. 63.04%, *p* < 0.001). Additionally, proteins related to the “Integrin family cell surface interactions” pathway were enriched in EVs from CTC-MCC-41.4 (36.21%, *p* < 0.001) and CTC-MCC-41.5G (34.4%, *p* = 0.017) cells (Fig. 3).

**Fig. 4:**
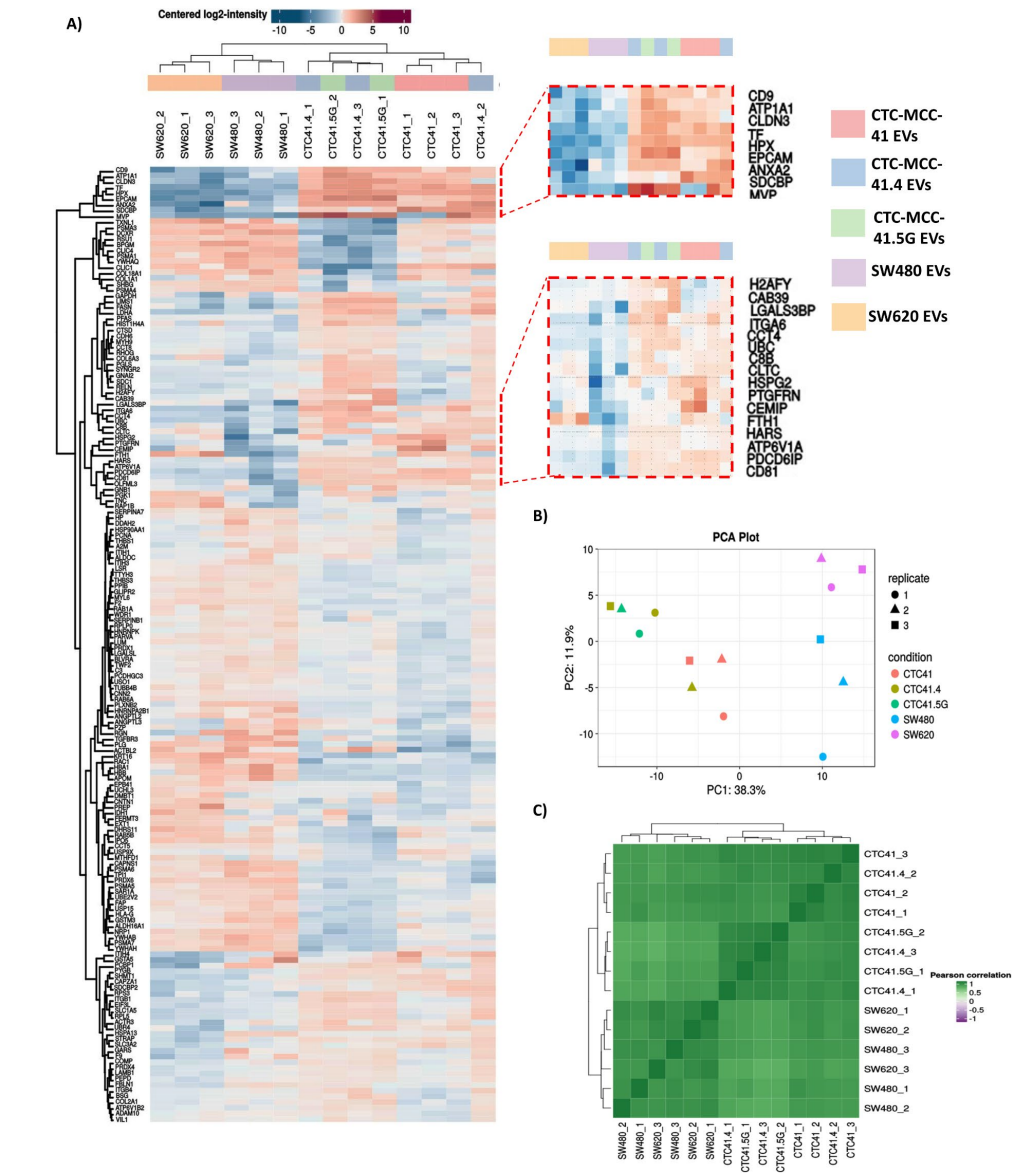
Label-free quantification analysis. (**A**) Heatmap showing the hierarchical clustering of the different EV preparations as a function of the differentially expressed proteins. CTC-derived EVs were separated from CRC cell-derived EVs, and EV samples were separated from the three CTC lines. **(B)** Principal component analysis showing clustering of the CTC-derived EV samples. CTC-MCC-41.4 and 41.5G clustered together in contrast to CTC-MCC-41, this in relation to their clonal status. **(C)** Correlation matrix showing a greater correlation between CTC lines

**Fig. 5:**
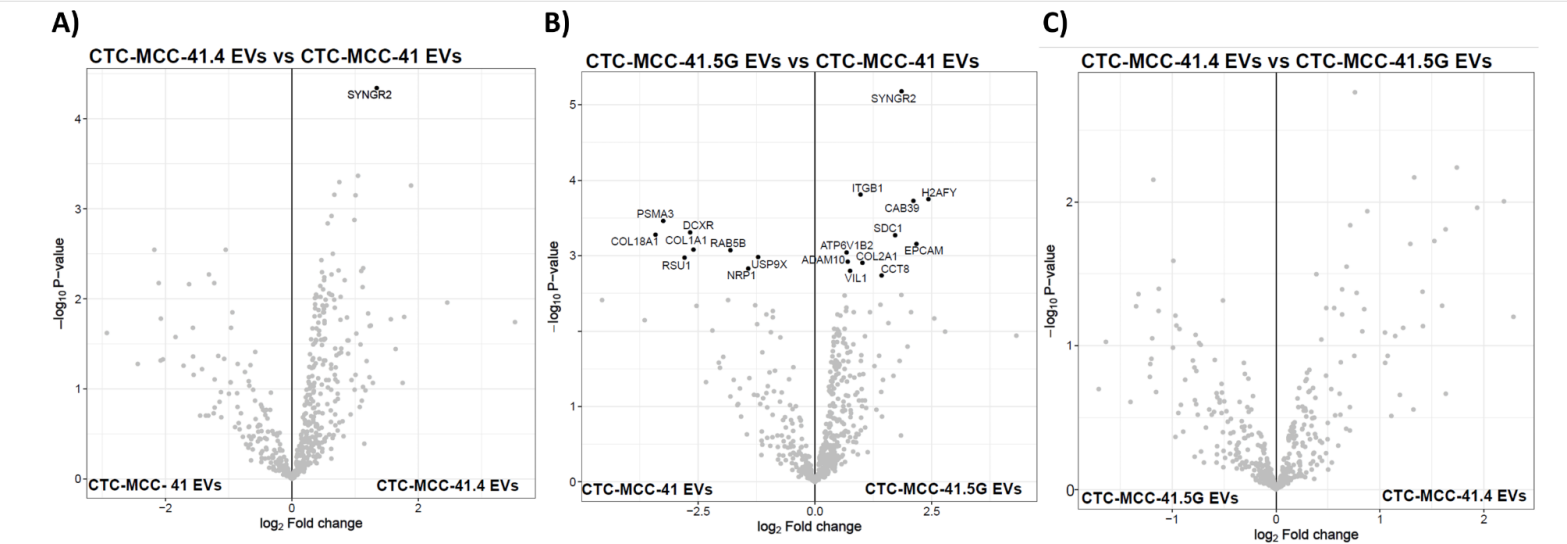
Volcano plots showing proteins that were differentially expressed between CTC line EV samples. (**A**) Comparison of EVs from CTC-MCC-41 and CTC-MCC-41.4 revealed that SYNGR2 was expressed at higher levels in CTC-MCC-41.4 EVs. (**B**) Comparison between EVs from CTC-MCC-41 and CTC-MCC-41.5G; PSMA3, DCXR, COL18A1, RSU1, RAB5B, USP9X, and NRP1 were upregulated in CTC-MCC-41 EVs, and SYNG2, ITGB1, CAB39, H2AFY, SDC1, EPCAM, ATP6V1B2, ADAM10, COL2A1, VIL1, and CCT8 were upregulated in CTC-MCC-41.5G EVs. Highlighting the differences in EVs cargo composition at different time points of progression represented by the CTC lines. (**C**) Comparison between EVs from CTC-MCC-41.5G and CTC-MCC-41.4; no differences were identified, reflecting their clonal similitude as both CTC lines represent the time point of progression despite therapy

Moreover, comparison of the number of proteins identified in the different EV samples by label-free quantitative (LFQ) mass spectrometry revealed that this number was the highest in CTC-MCC-41 EVs (Figure S2). In total, 168 proteins exhibited significant differences among the samples. Proteomic profiling revealed that CTC-derived EVs could be separated from SW480 and SW620 cell-derived EVs (Fig. 4a-c). Specifically, proteins of endosomal origin (e.g., CD9, CD81, PDCD6IP, and SDCBP) associated with EVs [15] were significantly more highly expressed in CTC-derived EVs, as were proteins related to organotropism and pre-metastatic niche formation (e.g., ITGA6, CEMIP, and ITGB1) [4, 5, 16]. Moreover, proteins of ectosomal origin were also significantly expressed in CTC-derived EVs (e.g., BSG and SLC3A2) [15], indicating that the three CTC lines released heterogeneous subpopulations of EVs (Figure S3). Moreover, EVs from CTC-MCC-41.4 and CTC-MCC-41.5G cells were more strongly correlated than EVs from CTC-MCC-41 cells were. This might be explained by their clonal status because the CTC-MCC-41 line was isolated before treatment initiation. Moreover, EVs from CTC-MCC-41 and CTC-MCC-41.5G cells presented significantly different protein expression profiles: PSMA3, DCXR, COL18A1, RSU1, RAB5B, USP9X, and NRP1 were upregulated in CTC-MCC-41 EVs, and SYNG2, ITGB1, CAB39, H2AFY, SDC1, EPCAM, ATP6V1B2, ADAM10, COL2A1, VIL1, and CCT8 were upregulated in CTC-MCC-41.5G EVs. Some of these proteins are associated with colorectal adenocarcinoma prognosis [17, 18] (Fig. 5a-c**)**.

**Fig. 6:**
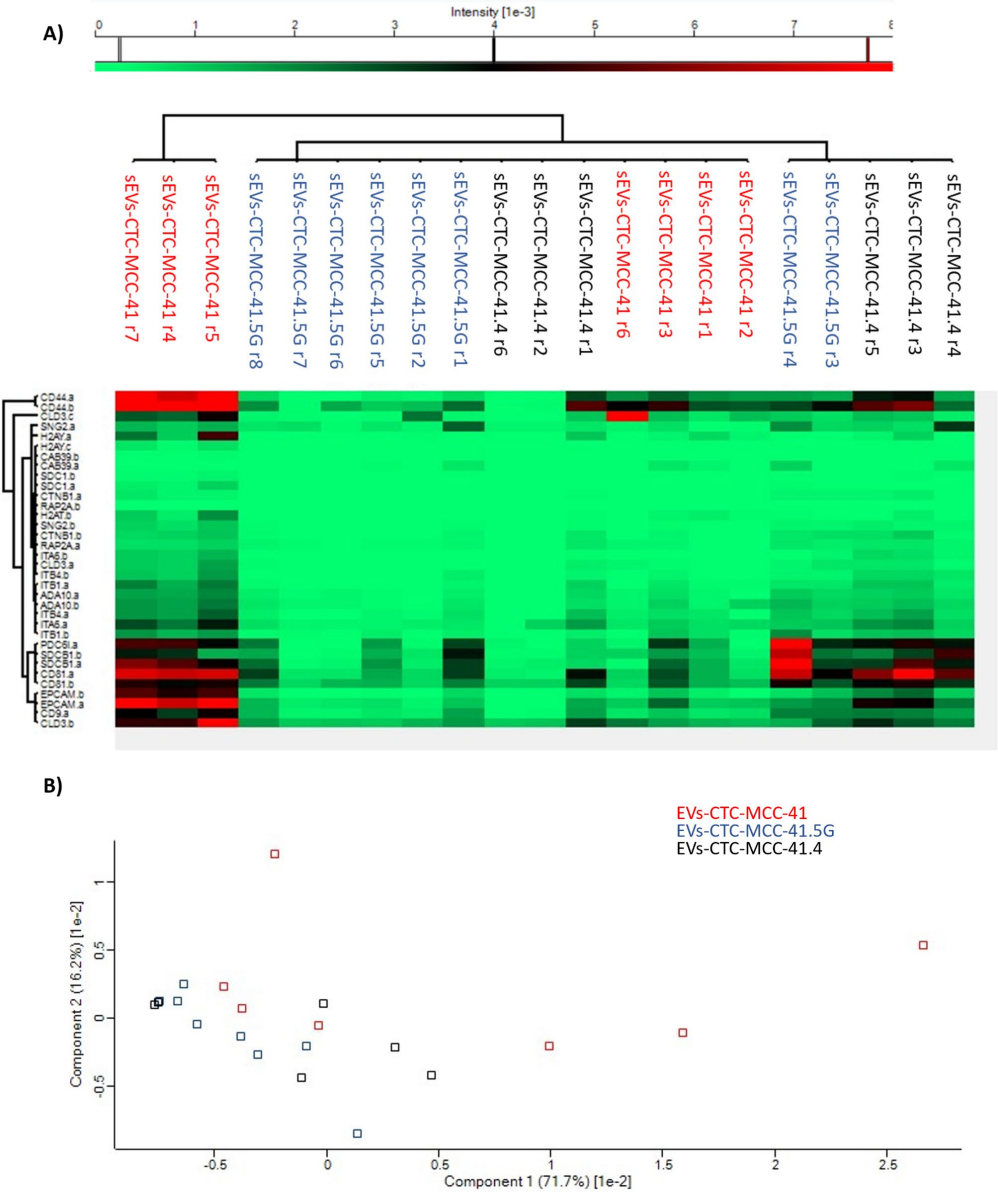
**(A)** Heatmap showing the hierarchical clustering of the different EV preparations as a function of selected peptides/proteins (MRM assay), highlighting sample heterogeneity between batches. **(B)** Principal component analysis showing high sample dispersion when the MRM data were used. A clear grouping classification was not possible

On the basis of the LFQ results (Figure S4), relevant proteins (pivotal for CRC biology, metastasis, and EV biogenesis) were selected to confirm their presence in CTC-derived EVs via an MRM assay: CD9, CD81, CD44, CLD3, CTNNB1, EPCAM, ITGA6, ITGB1, ITGB4, PDCD6IP, RAP2B, SDCP, SYNGR2, ADAM10, CAB39, and H2AFY. Their presence was confirmed. However, considerable variability was observed when this method was used to detect individual proteins. Moreover, EV classification via principal component analysis was not feasible when the MRM data were used (Fig. 6).

### Biodistribution of CTC-derived EVs in NOD-SCID mice

**Fig. 7:**
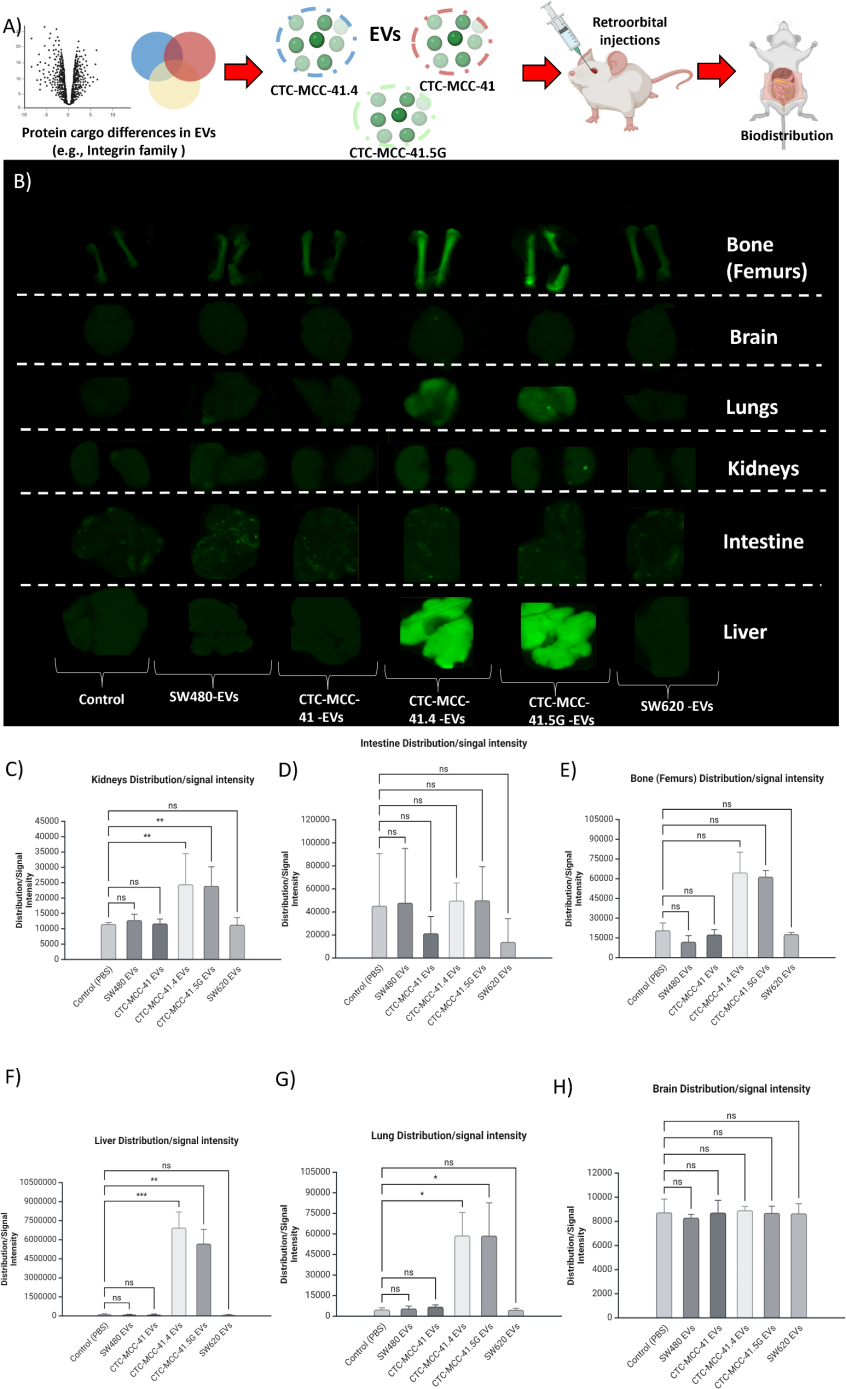
**(A)** Schematic representation of the experimental design. Protein cargo differences were identified in EVs from the different cell lines, then labeled EVs were injected retro-orbitally into NOD-SCID mice for each cell line; finally, EV organ distribution was analyzed after 24 h. **(B)** Representative images of EV uptake in the femur, brain, lungs, kidneys, intestine and liver. **C to H)** Mean signal intensity distributions. The uptake of EVs from CTC-MCC-41.4 and 41.5G cells was significantly greater in the kidneys, femurs, liver and lungs than in the other colon cancer cell lines and the control (PBS). EVs from CTC-MCC-41.4 and 41.5G preferentially accumulate in the liver and lungs, indicating higher potential for metastasis that might be associated to the time-point of progression despite therapy (One way ANOVA test in panels F [*p* < 0.0001], G [*p* = 0.000332] and H [*p* = 0.9174]; Kruskal‒Wallis test in panels C [*p* = 0.00154], D [*p* = 0.171], and E [*p* = 0.00062]; *P* < 0.01 for significance) (*post hoc* analysis in panels C, D, E, F, G, and H, *P < 0.05, ** *P* < 0.01, ***P < 0.001, ns = not significant)

To investigate whether the differences in EV proteome cargo between cell lines affect organ distribution (i.e., biodistribution), due to higher proportion of proteins associated to the integrin family cell surface interactions in EVs of CTC-MCC-41.4 and CTC-MCC-41.5G. EVs were labeled with a far-red fluorochrome and retro-orbitally injected into NOD-SCID mice (Fig. 7a). Imaging of the liver, kidneys, brain, intestine, lungs and femurs after 24 h revealed that EV biodistribution and uptake varied depending on the cell line from which they were derived **(**Fig. 7b**)**. EVs from CTC-MCC-41.4 and CTC-MCC-41.5G cells significantly accumulated in the liver, followed by the lungs, kidneys and femurs, compared with the other EV samples and the control (PBS) **(**Fig. 7c, e, g**)**. Conversely, EVs were not detected in the brain or intestine (Fig. 7d, h).

## Discussion

Changes in the EV proteome during cancer progression represent an opportunity to elucidate metastasis mechanisms [7]. In this study, we investigated the proteomic profiles of EVs derived from three colon metastasis-competent CTC lines representing the clonal evolution of CRC during therapy. We identified several proteins that were differentially expressed in EVs from the CTC lines obtained after therapy initiation, indicating changes, adaptations and alterations associated with tumor/CTC clone resistance to standard chemotherapy. These colon CTC lines also present distinct metabolic profiles [2].

The size-based characterization and morphology results indicated that EVs were predominant in our preparations. The highest secretion of particles per milliliter and highest protein content (micrograms per milliliter) were observed with the CTC-MCC-41.5G line derived after the third relapse (liver metastases) following progression during therapy [2]. This observation suggested that changes in EV secretion were related to the clonal status of the CTC lines.

EV characterization on the basis of their proteomic profile revealed heterogeneity among EV subpopulations. EVs were positive for the CD9 and CD81 markers but not for CD63. The absence of CD63 might suggest that EVs released by CTC lines do not conform to the classical definition of “exosomes” [19]. However, CD63 is not always present in EVs, and Hoshino et al. proposed that alternative EV markers should be considered [14], highlighting the frequent heterogeneity in EV populations. In their study, CD63 was identified only in EVs from murine tissue and not from human plasma or tissue samples. Moreover, the role of CD63 in identifying EVs as exosomes is still debatable. Additionally, comparison of the proteins present in our EV preparations with the most commonly reported EV-associated proteins (e.g., Vesiclepedia) and those identified by Hoshino et al. (2020) suggested an enrichment of exosomes in CTC-derived EVs compared with CRC cell-derived EVs. These differences between CTC-derived and CRC cell-derived EVs highlight the importance of comparing different cancer cell lines and CTC lines from the same patient at different stages of disease progression to confirm that these differences are due to intrinsic biological differences and not to technical and analytical variables.

A comparison of EVs from CTC-MCC-41 and CTC-MCC-41.5G cells (obtained before and after therapy failure, respectively) revealed a significantly greater presence of proteins associated with stemness (CD44 and EpCAM) [7, 20] and proteins implicated in prognosis and cancer metastasis (ITGB1, CAB39, H2AFY, VIL1, CCT8, and ADAM10) [17, 18]. For instance, VIL1 is associated to proliferation and migration in CRC, similarly to ADAM10 and ITGB1 [21–23]. In another hand, CCT8 expression is related to worse overall survival in CRC [24], and CAB39 is associated to chemoresistance pathways [17]. Nevertheless, the role of these proteins in CRC progression is still not fully understood in the clinical context, our results show that there is potential for their evaluation using EVs in blood circulation of CRC patients.

The higher SYNGR2 signal in CTC-MCC-41.5G EVs suggests the presence of microvesicles, another EV type, again showing the heterogeneity of EV subpopulations that is evident only when proteomic approaches are used, unlike the characterization performed using a few selected markers. These differences were not significant between CTC-MCC-41.4 EVs and the other two types of CTC-derived EVs but were only significant when they were compared with SW480 and SW620 cell-derived EVs.

Principal component analysis (PCA) and hierarchical classification (heatmaps) to identify protein signatures of EVs derived from different cell lines revealed that EVs derived from CTC-MCC-41.4 and CTC-MCC-41.5G cells clustered together (i.e., the two CTC lines derived after therapy failure). Moreover, all CTC-derived EVs were classified separately from SW480 and SW620 cell-derived EVs, highlighting a CTC-specific EV cargo, which is different from that of EVs released by primary and metastatic CRC cells. This was supported by the correlation matrix. Differences between EVs from CTC lines and those from other CRC cell lines might be due to culture conditions or genomic differences. The distinct profile of proteins packed in EVs from CTC-MCC-41.5G cells suggests that these proteome changes are linked to cancer progression and more aggressive clones. Indeed, the CTC-MCC-41.5G line is more aggressive because it originates after CRC spreads to the liver (more advanced clonal selection). It could be hypothesized that EVs from this line display a different cargo and therefore a different organotropism and greater metastatic potential.

Our work focused primarily on differences in EVs from CTC lines, whereas other studies compared SW480 and SW620 cell-derived EVs. For example, Choi et al. reported that changes in the proteome composition of EVs derived from these two CRC cell lines were correlated with progression because the SW620 cell line was obtained from a metastatic site. In our analysis, the same changes in EVs from these cell lines were not detected, possibly due to differences in EV isolation protocols [25, 26].

Additionally, a limitation of LFQ data is the use of imputation methods to handle missing values. These methods might introduce bias in the identification of the proteins packed in EVs [27]. Therefore, in this study, the presence of relevant proteins was confirmed via targeted proteomics via an MRM panel. This panel of peptides showed high variations in the protein compositions of EV samples, highlighting the limitation of using single protein markers for EV characterization. Different EV preparations did not show similar clustering as the LFQ approach but tended to form subclusters related to the CTC lines. However, different amounts of proteins in EVs from in vitro culture might reflect different cell culture conditions and variations in protein quantification because the samples were isolated and processed at different time-points. Further standardization is required to classify EVs on the basis of this panel.

The next step was to determine whether their different proteome profiles influenced EV biodistribution in vivo. The same EV batches were used for the proteomic analyses and for the biodistribution assay to directly correlate the biodistribution and proteome data and reduce the bias of culture conditions that might influence EV secretion. EVs derived from CTC-MCC-41.4 and CTC-MCC-41.5G cells exhibited distinct distribution patterns, possibly due to their “exosomal” profile, according to Vesiclepedia [13], and their greater abundance of surface proteins associated with metastasis (ITGB1, CD44, and EpCAM). These surface proteins might promote EV extravasation and tissue attachment. Notably, even slight variations in protein quantities between EVs from the CTC lines may have a significant impact on their function. This distribution pattern could also be explained by the presence of other particles, such as exomeres, in our preparations. EV uptake by the lungs, liver and femur has already been described by other groups, whereas its distribution to the kidney is not well understood. EVs might have a role in kidney xenobiotic metabolism, such as exomeres in the liver; however, this role must be investigated in future studies.

If EV secretion and release are linked to mechanisms of cancer progression and therapy resistance, they could provide a more precise method for monitoring cancer progression than changes in tumor size, which remains the gold standard for evaluating CRC progression. This is because tumor growth is a consequence rather than the cause of therapy failure. However, a key limitation of using EVs as biomarkers in blood-based “liquid biopsy” is the presence of physiological EVs and lipoproteins, which hinder the specific detection of tumor-associated EVs and restrict the clinical utility of bulk protein detection methods such as Western-blot or LFP. In this study, we demonstrated the potential of alternative proteomic approaches, such as MRM “Targeted proteomics”, to detect relevant CRC-EV-associated proteins based on specific peptides identified. This strategy could enhance the accuracy of detecting tumor-associated EVs in the blood circulation of CRC patients.

## Conclusion

This study is the first to describe the proteomic composition of EVs derived from metastasis-competent CTCs. These CTC lines are the only model that mirrors disease progression in patients with mCRC despite therapy. The present findings provide strong evidence that changes in EV cargo are associated with clonal evolution during the development of therapy resistance. These results provide *(i)* a better understanding of the role of EVs in cancer and *(ii)* potential applications in liquid biopsy-based methods for the complementary diagnosis of disease progression in response to therapy. Nevertheless, further in vivo studies are needed to validate these initial findings. For example, different CRC subtypes may exhibit distinct profiles of EV-associated proteins. Additionally, more experiments exposing CTC lines to chemotherapy agents are required to confirm how EV cargo dynamically changes in response to treatment.

**Fig. 8:**
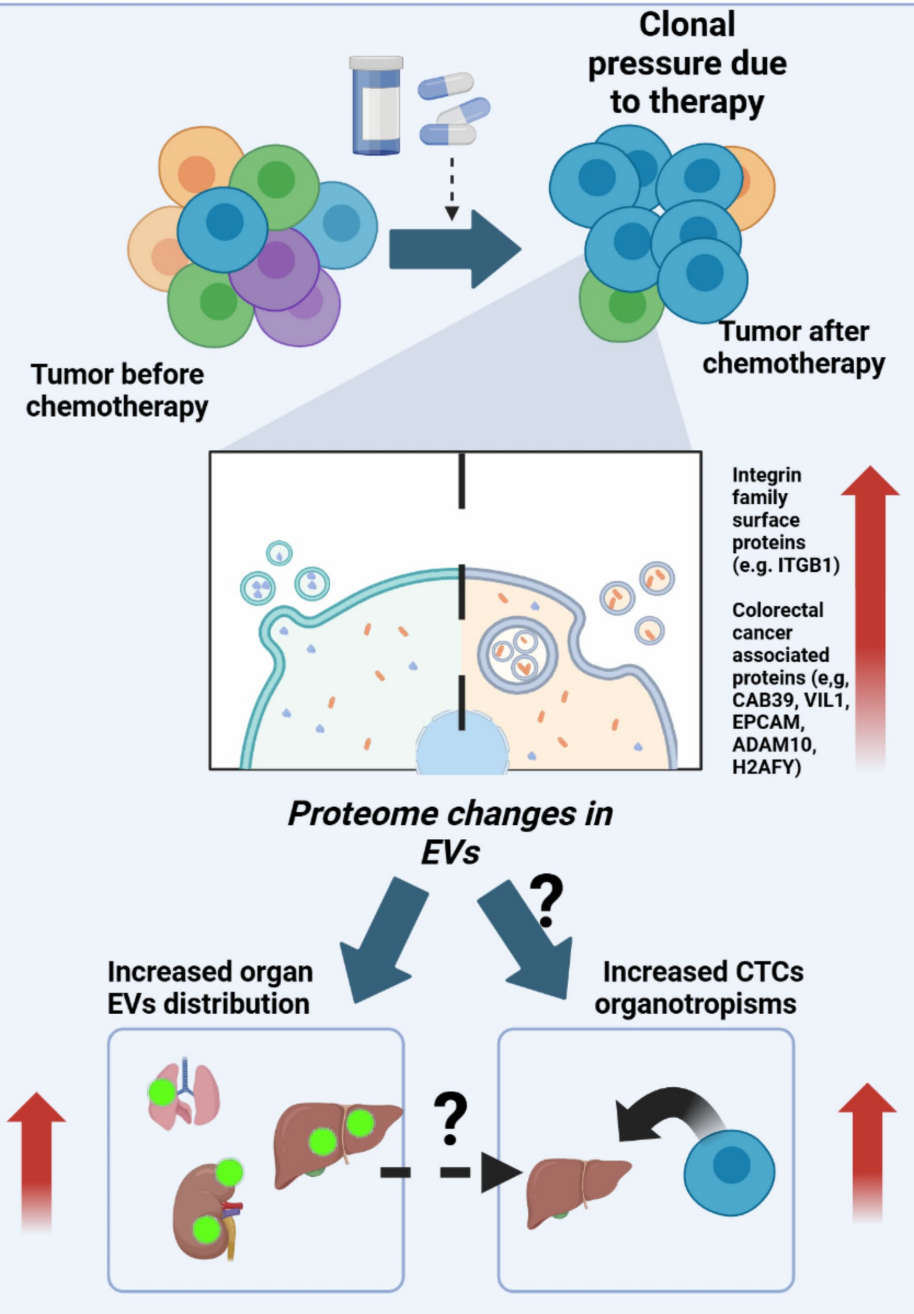
Proposed mechanism for the role of EVs in cancer progression. Clonal pressure due to chemotherapy or other cancer therapies might influence the proteome composition of EVs. These proteomic changes might influence EV distribution and CTC organotropism, ultimately increasing the risk of metastasis

We have shown that an increased presence of proteins associated with the integrin family is found in EVs from CTC lines obtained after therapy failure, and these EVs show increased affinity for organs such as the liver and lungs. It is essential to determine if this have an effect on the organotropism of CTCs in vivo; and whether these EV alterations are also present in patients with CRC. For example, the use of MRM panels to analyze EVs from plasma samples might reveal a more complex proteome in CRC (see Fig. 8).

Given the need for analytical methods to support cancer progression diagnosis, functional circulating biomarkers, such as EVs, could provide valuable insights, particularly when radiological examination is limited. In this context, our findings suggest that therapy-driven clonal evolution influences the protein cargo of EVs associated with CRC progression and modulates the presence of surface proteins (e.g., integrins), which play a key role in pre-metastatic niche formation and organotropism.

## Electronic supplementary material

Below is the link to the electronic supplementary material.

Supplementary Material 1

## Data Availability

The datasets supporting the conclusions of this article are available in the ProteomeXchange Consortium via the PRIDE partner repository, identifier PXD055639.

## Abbreviations

ABC: Ammonium bicarbonate
CRC: Colorectal cancer
CTC: Circulating tumor cells
DTT: Dithiothreitol
EVs: Extracellular vesicles
FBS: Fetal bovine serum
FDR: False detection rate
IAA − 2: Iodoacetamide
ISEV: International Society of Extracellular Vesicles
LFQ: Label-free quantification
mCRC: Metastatic colorectal cancer
MRM: Multiple reaction monitoring
NTA: Nanotracking analysis
PBS: Phosphate-buffered saline
5-FU: 5-Fluorouracil
