# Correction: Proteomic profiling and functional analysis of extracellular vesicles from metastasis-competent circulating tumor cells in colon cancer

**DOI:** 10.1186/s13046-025-03384-w

**Published:** 2025-04-09

**Authors:** Luis Enrique Cortés-Hernández, Zahra Eslami-S, Aurore Attina, Silvia Batista, Laure Cayrefourcq, Jérôme Vialeret, Dolores Di Vizio, Christophe Hirtz, Bruno Costa-Silva, Catherine Alix-Panabières

**Affiliations:** 1 Laboratory of Rare Human Circulating Cells, University Medical Center of Montpellier, Montpellier, France; 2 CREEC, MIVEGEC, University of Montpellier, CNRS, IRD, Montpellier, France; 3 IRMB-PPC, INM, Univ Montpellier, CHU Montpellier, INSERM CNRS, Montpellier, France; 4 Systems Oncology Group, Champalimaud Centre for the Unknown, Lisbon, Portugal; 5 Department of Urology, Division of Cancer Biology and Therapeutics, Cedars-Sinai Medical Center, Los Angeles, CA USA; 6 European Liquid Biopsy Society (ELBS), Hamburg, Germany

**Correction** **:** **J Exp Clin Cancer Res 44, 102 (2025)**

**https://doi.org/10.1186/s13046-025–03360- 4**

Following the publication of the original article [1], the authors identified an error in Figure 2. The current Figure 2 is a duplication of Figure 5. The figure is now being corrected.

**Incorrect **Fig. 2

**Fig. 2:**
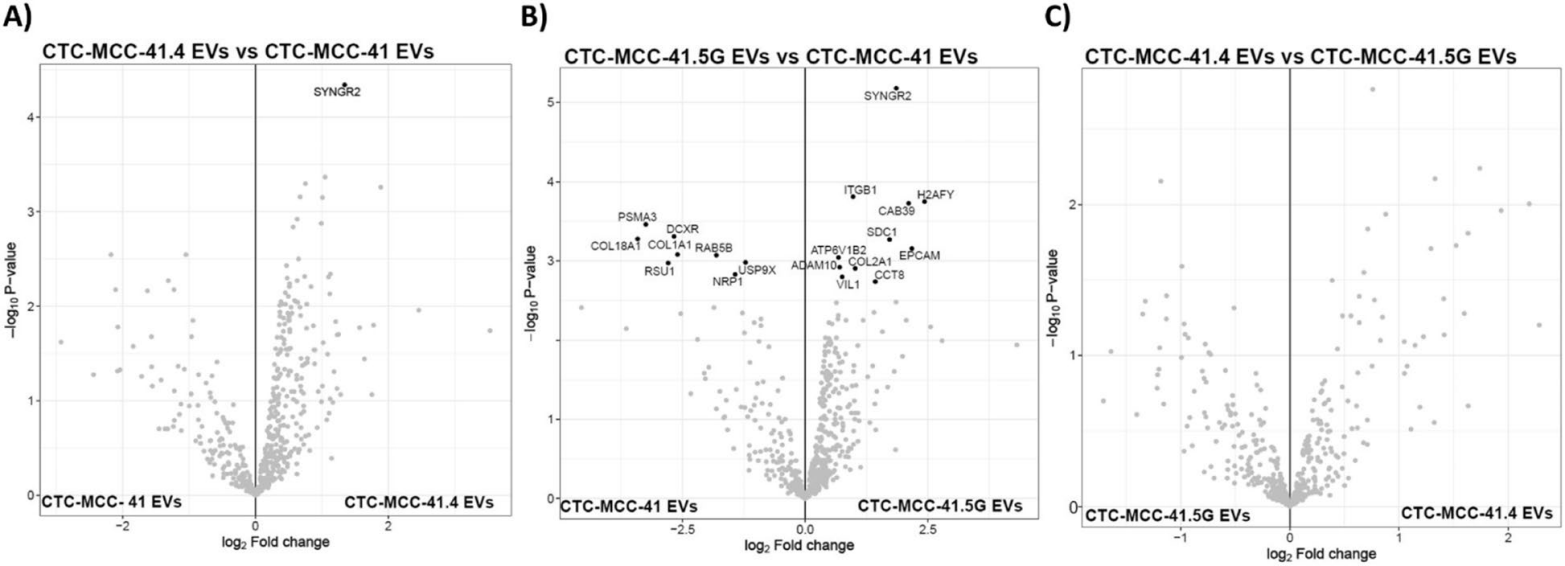
Cellular component analysis of the EV proteomic cargo based on present-absent analysis. **A**) to **E**) Comparison of the EV proteins identified with those listed in Vesiclepedia and those identified by Hoshino et al. (2020). **F**) ANXA5 was not detected in EV preparations from the three CTC lines or the two colon cancer cell lines, and CD9 was absent only in SW480 and SW620 cell-derived EVs. **G**) Cellular component enrichment in the indicated EV samples according to the Vesiclepedia data for “Cytoplasm” and “Exosomes”, revealing significantly greater enrichment of “Exosome”-associated proteins in EVs from CTC-MCC- 41.4 and CTC-MCC- 41.5G cells (derived after therapy failure) than in those from the other colon cancer cell lines

**Correct** Fig. 2

**Fig. 2:**
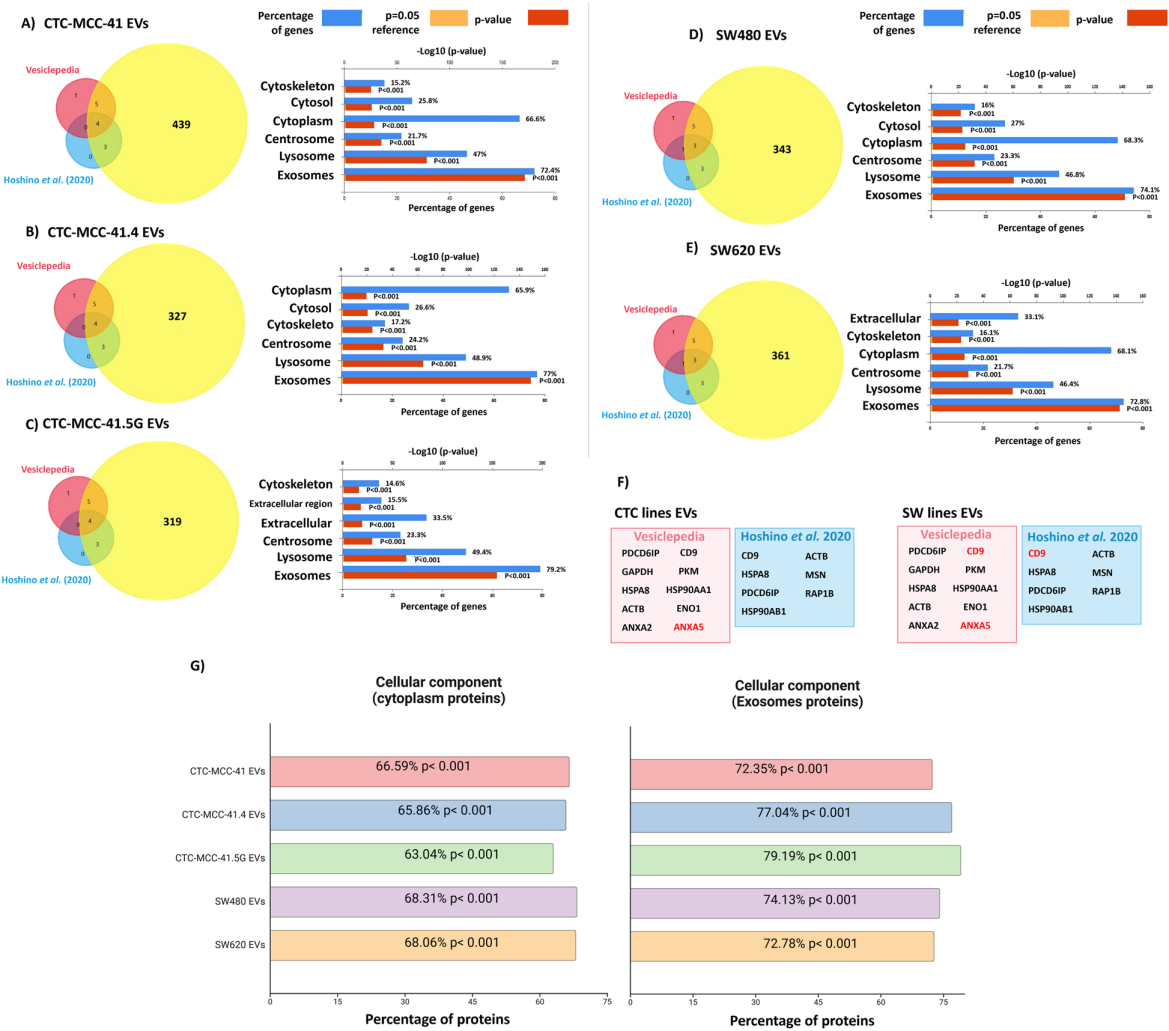
Cellular component analysis of the EV proteomic cargo based on present-absent analysis. **A**) to **E**) Comparison of the EV proteins identified with those listed in Vesiclepedia and those identified by Hoshino et al. (2020). **F**) ANXA5 was not detected in EV preparations from the three CTC lines or the two colon cancer cell lines, and CD9 was absent only in SW480 and SW620 cell-derived EVs. **G**) Cellular component enrichment in the indicated EV samples according to the Vesiclepedia data for “Cytoplasm” and “Exosomes”, revealing significantly greater enrichment of “Exosome”-associated proteins in EVs from CTC-MCC- 41.4 and CTC-MCC- 41.5G cells (derived after therapy failure) than in those from the other colon cancer cell lines

The correction does not compromise the validity of the conclusions and the overall content of the article. The original article [1] has been corrected.
