# Supplementary material for: Proteomic profiling and functional analysis of extracellular vesicles from metastasis-competent circulating tumor cells in colon cancer
Source: J Exp Clin Cancer Res. 2025 Mar 22;44:102. doi: 10.1186/s13046-025-03360-4 (PMC11929255; doi:10.1186/s13046-025-03360-4)
Supplement: Supplementary file 1 — Supplementary Material 1 [file 13046_2025_3360_MOESM1_ESM.docx]

**Supplementary**

| Proteins | Peptides |
| --- | --- |
| CD44_HUMAN | R.YGFIEGHVVIPR.I |
| CD44_HUMAN | K.LVINSGNGAVEDR.K |
| CD81_HUMAN | K.QFYDQALQQAVVDDDANNAK.A |
| CD81_HUMAN | K.TFHETLDCCGSSTLTALTTSVLK.N |
| CD9_HUMAN | K.DVLETFTVK.S |
| CD9_HUMAN | K.AIHYALNCCGLAGGVEQFISDICPK.K |
| CLD3_HUMAN | K.VYDSLLALPQDLQAAR.A |
| CLD3_HUMAN | R.DFYNPVVPEAQK.R |
| CLD3_HUMAN | R.STGPGASLGTGYDR.K |
| CTNB1_HUMAN | R.GLNTIPLFVQLLYSPIENIQR.V |
| CTNB1_HUMAN | R.NEGVATYAAAVLFR.M |
| EPCAM_HUMAN | R.AKPEGALQNNDGLYDPDCDESGLFK.A |
| EPCAM_HUMAN | K.TQNDVDIADVAYYFEK.D |
| ITA6_HUMAN | K.LIATFPDTLTYSAYR.E |
| ITA6_HUMAN | K.SEDEVGSLIEYEFR.V |
| ITB1_HUMAN | K.LKPEDITQIQPQQLVLR.L |
| ITB1_HUMAN | K.LSENNIQTIFAVTEEFQPVYK.E |
| ITB4_HUMAN | R.VLSQLTSDYTIGFGK.F |
| ITB4_HUMAN | K.NVISLTEDVDEFR.N |
| RAP2A_HUMAN | K.VVVLGSGGVGK.S |
| RAP2A_HUMAN | K.YDPTIEDFYR.K |
| SDC1_HUMAN | K.EGEAVVLPEVEPGLTAR.E |
| SDC1_HUMAN | K.DEGSYSLEEPK.Q |
| SDCB1_HUMAN | K.SIDNGIFVQLVQANSPASLVGLR.F |
| SDCB1_HUMAN | R.NGLLTEHNICEINGQNVIGLK.D |
| SNG2_HUMAN | R.FLTQPQVVAR.A |
| SNG2_HUMAN | K.DVLVGADSVR.A |
| ADA10_HUMAN | K.AIDTIYQTTDFSGIR.N |
| ADA10_HUMAN | R.TITLQPGSPCNDFR.G |
| CAB39_HUMAN | K.DVAQIFNNILR.R |
| CAB39_HUMAN | K.LLGELLLDR.H |
| H2AY_HUMAN | K.AGVIFPVGR.M |
| H2AY_HUMAN | R.HILLAVANDEELNQLLK.G |
| H2AY_HUMAN | K.SIAFPSIGSGR.N |
| PDC6I_HUMAN | R.SVIEQGGIQTVDQLIK.E |
| PDC6I_HUMAN | K.FYNELTEILVR.F |

**Table S1**. **Peptides selected for the MRM assay**. List of peptides selected for MRM. CD9-K.AIHYALNCCGLAGGVEQFISDICPK. K was discarded because of a lack of detection.


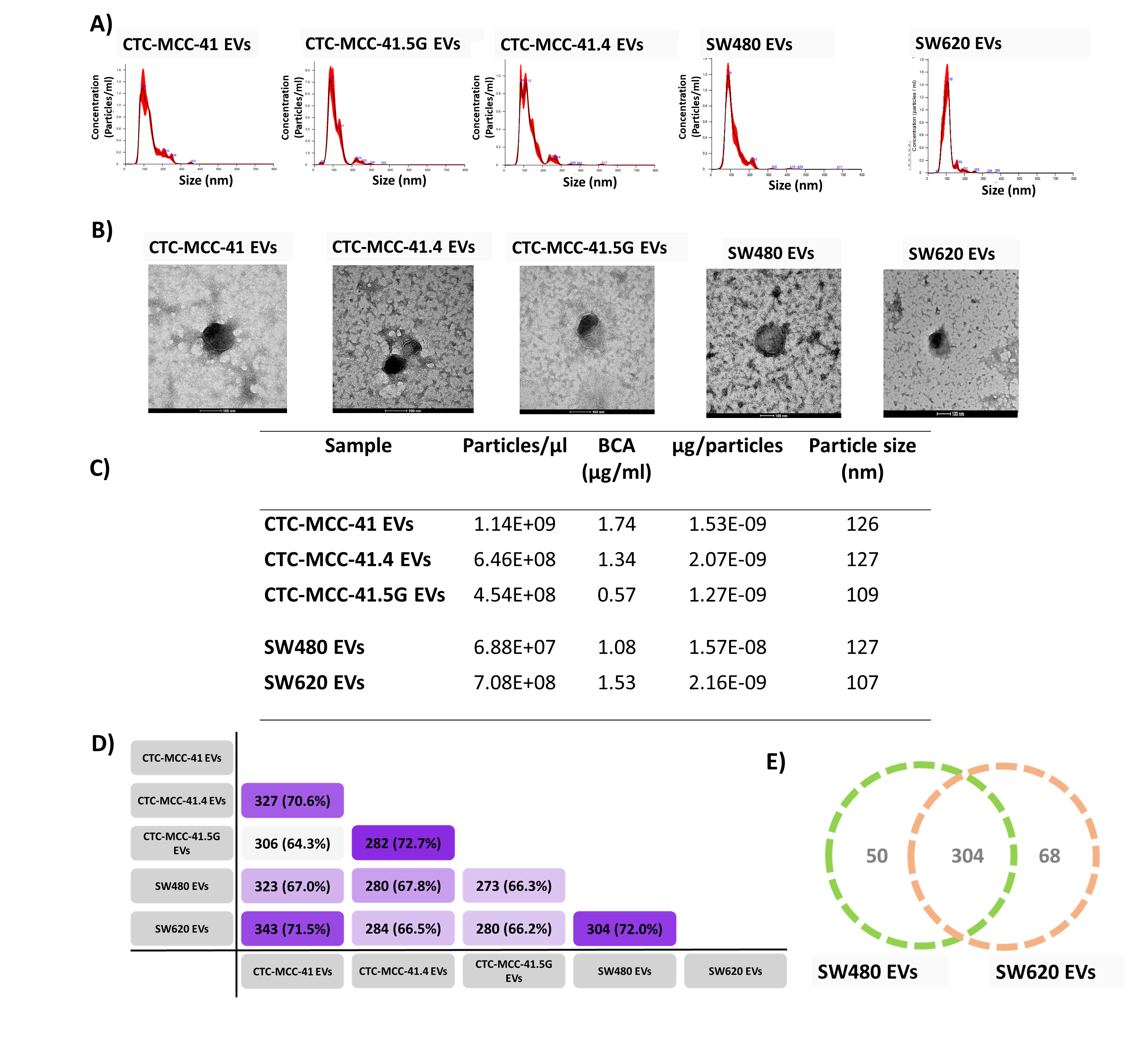


**Figure S1. EV characterization. A)** Particle analysis showing the enrichment of particles with a size compatible with EVs, particularly in CTC-MCC-41.5G cell-derived samples. **B)** Representative electron microscopy images showing the classical “cup-shaped” morphology that reflects the vesicular features of the isolated samples**. C)** General description of the particles and total proteins in the samples that were used for the proteomic analysis and biodistribution assays. **D)** Percentages of proteins shared by the different EV samples. **E)** Venn diagram showing the number of shared and unique proteins in EVs from SW480 and SW620 cells.


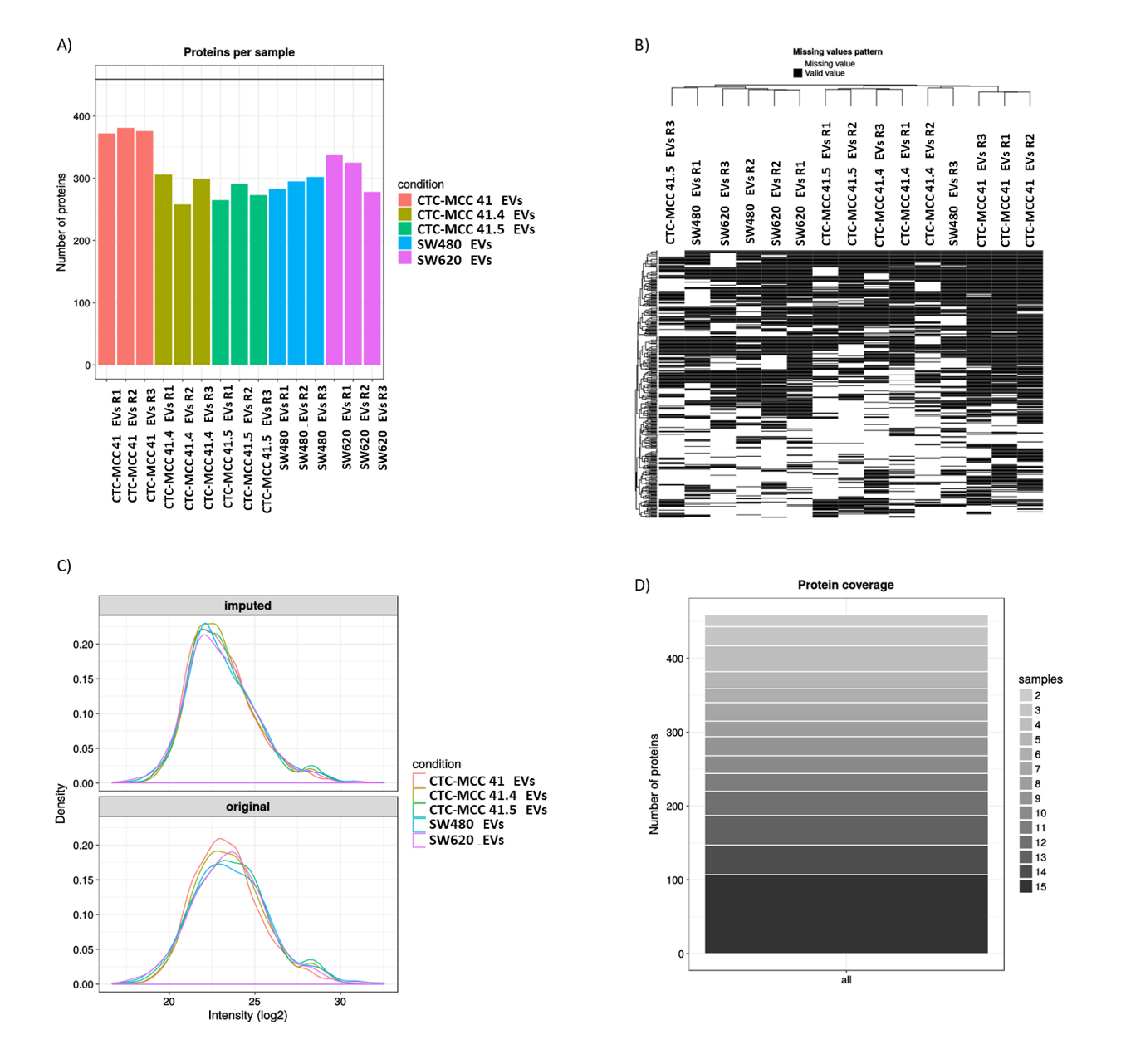


**Figure S2.** **A)** Total number of proteins per EV sample detected by LFQ. CTC-MCC-41 EVs presented the greatest number of proteins detected. **B)** Heatmap of the missing value patterns per sample. **C)** Imputation method (BPCA); this method introduces bias at lower intensities. **D)** Protein coverage by number of samples; all samples shared at least >100 proteins.


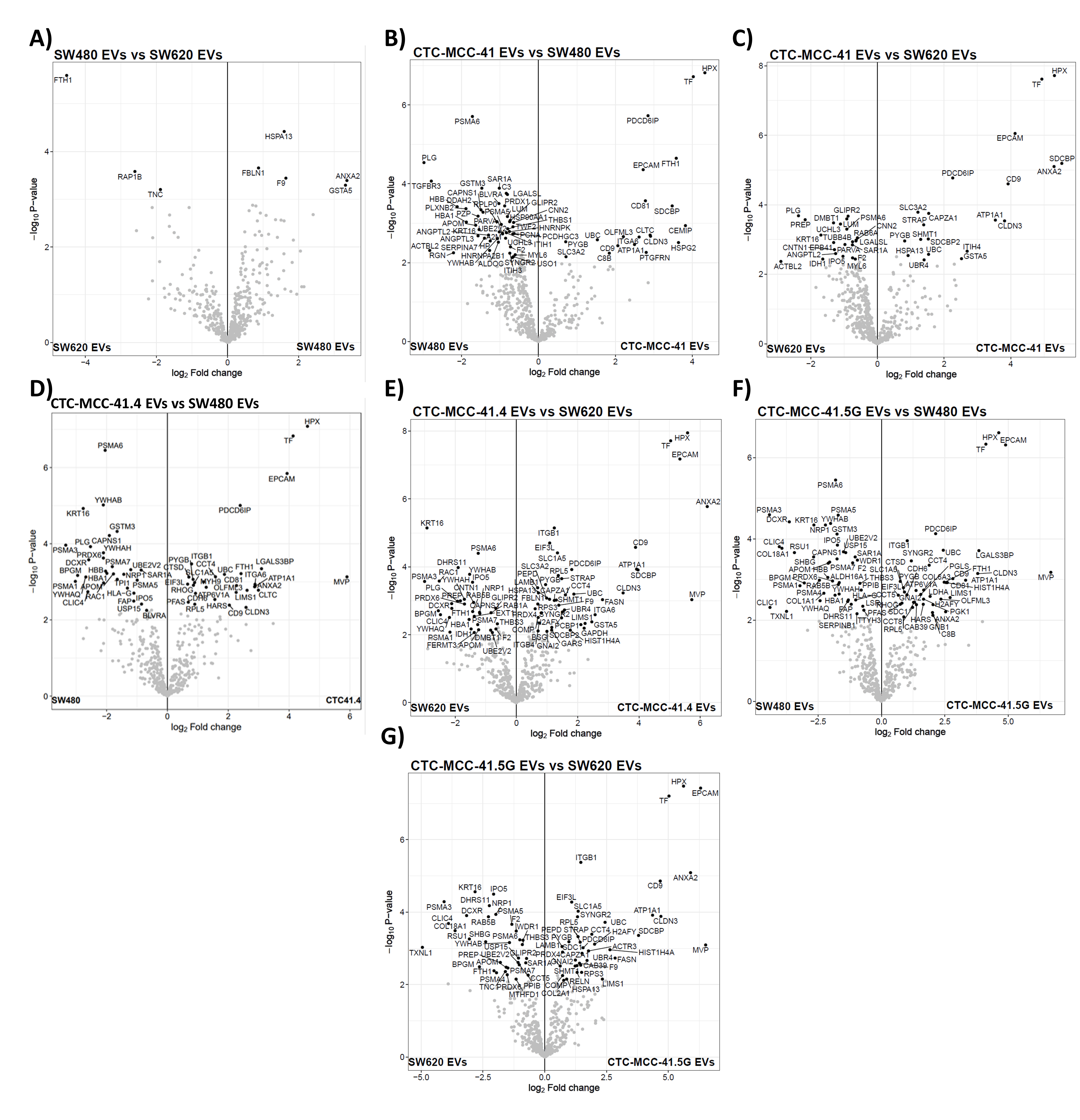


**Figure S3.** Volcano plots showing proteins that were differentially expressed between CTC line-derived EVs, SW480-derived EVs and SW620-derived EVs. A) Comparison between SW480 EVs and SW620 EV “colon cancer cell lines”. B-C) Comparison between CTC-MCC-41 EVs and colon cancer cell lines. D-E) Comparison between CTC-MCC-41.4 EVs and colon cancer cell lines. F-G) Comparison between CTC-MCC-41.5G EVs and colon cancer cell lines.


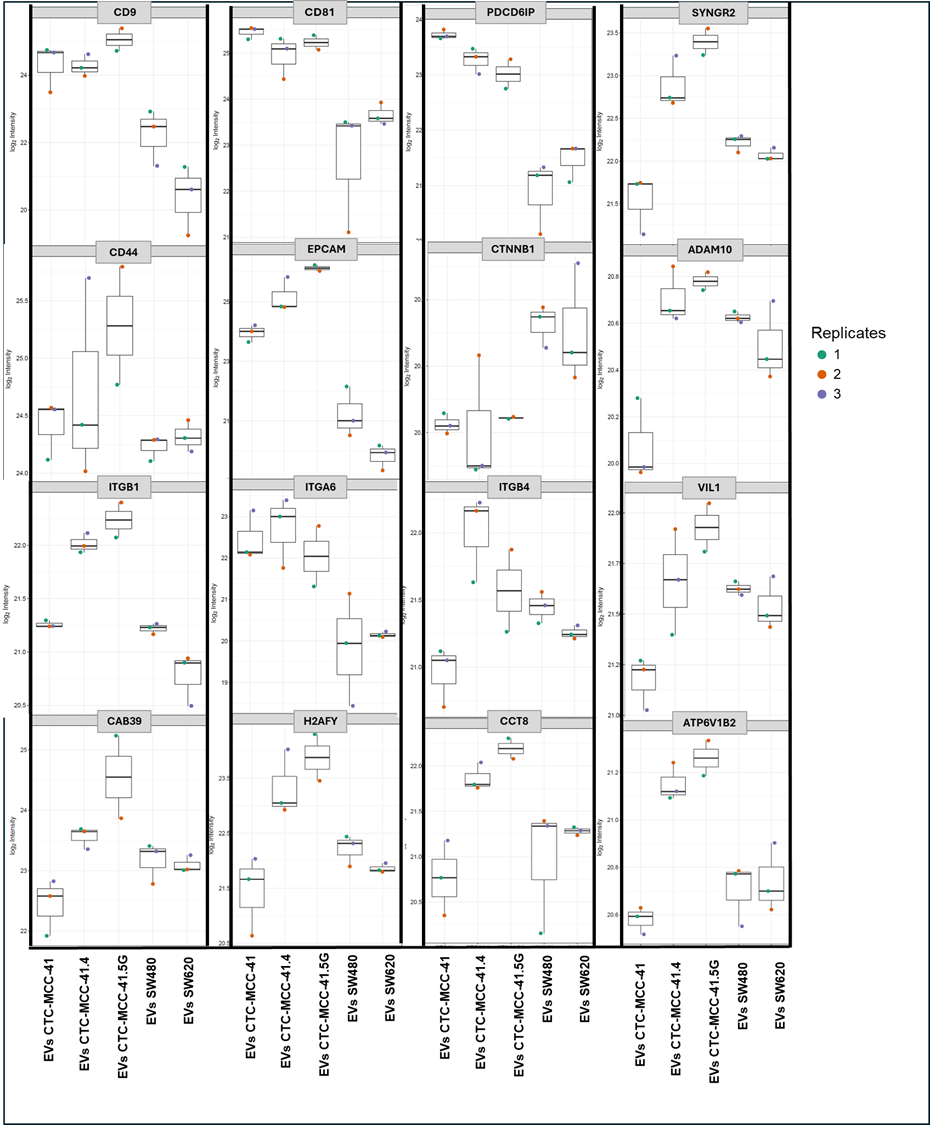


**Figure S4**. LFQ intensities of CD9, CD81, PDC6IP, SYNGR2, CD44, EPCAM, CTNNB1, ADAM10, ITGB1, ITGA6, ITGB4, VIL1, CAB39, H2AFY, CCT8 and ATP6V1B2 in the indicated EV samples.
